# Comparison of Methods for Estimating Prevalence of Chronic Diseases and Health Behaviors for Small Geographic Areas: Boston Validation Study, 2013

**DOI:** 10.5888/pcd14.170281

**Published:** 2017-10-19

**Authors:** Yan Wang, James B. Holt, Xingyou Zhang, Hua Lu, Snehal N. Shah, Daniel P. Dooley, Kevin A. Matthews, Janet B. Croft

**Affiliations:** 1 Division of Population Health, National Center for Chronic Disease Prevention and Health Promotion, Centers for Disease Control and Prevention, Atlanta, Georgia; 2 Economic Research Service, US Department of Agriculture, Washington, District of Columbia; 3 Boston Public Health Commission, Boston, Massachusetts; 4 Boston University, School of Medicine, Boston, Massachusetts

## Abstract

**Introduction:**

Local health authorities need small-area estimates for prevalence of chronic diseases and health behaviors for multiple purposes. We generated city-level and census-tract–level prevalence estimates of 27 measures for the 500 largest US cities.

**Methods:**

To validate the methodology, we constructed multilevel logistic regressions to predict 10 selected health indicators among adults aged 18 years or older by using 2013 Behavioral Risk Factor Surveillance System (BRFSS) data; we applied their predicted probabilities to census population data to generate city-level, neighborhood-level, and zip-code–level estimates for the city of Boston, Massachusetts.

**Results:**

By comparing the predicted estimates with their corresponding direct estimates from a locally administered survey (Boston BRFSS 2010 and 2013), we found that our model-based estimates for most of the selected health indicators at the city level were close to the direct estimates from the local survey. We also found strong correlation between the model-based estimates and direct survey estimates at neighborhood and zip code levels for most indicators.

**Conclusion:**

Findings suggest that our model-based estimates are reliable and valid at the city level for certain health outcomes. Local health authorities can use the neighborhood-level estimates if high quality local health survey data are not otherwise available.

## Introduction

Local governments need measures of population health at the level of small geographic areas for multiple purposes, such as planning public health prevention programs, allocating resources, formulating health policy, and health care decision making and delivery. However, little population health survey data exist at the county and subcounty levels. Although various national health surveys are available, such as the Behavioral Risk Factor Surveillance System (BRFSS) and the National Health Interview Survey (NHIS), direct estimates of population health measures are designed to be representative of the population at the state level (BRFSS) or larger regions (NHIS); direct estimates for small areas below the state level often are less reliable because of limited coverage or small sample sizes in the small areas that are covered (1,2). To obtain public health data at the small-area level, different approaches, including model-based estimation techniques, have been developed to produce local estimates of various chronic diseases and health-related behaviors (3–6). One such method is a multilevel model that includes area-specific random effects to account for between-area variations; this method has been shown to produce more valid and precise county-level estimates than other methods (7,8).

We previously applied a multilevel regression model and post-stratification (MRP) method using BRFSS data to estimate the prevalence of chronic health conditions and behaviors at multiple geographic levels (9). In brief, we constructed a multilevel logistic model and applied it to make predictions using US Census 2010 population counts at the smallest geographic level (the census block) that could be further aggregated to produce reliable health-indicator estimates at other geographic levels of interest. By comparing estimates generated by our model with direct county-level estimates from local surveys, such as the 2011 Missouri County-Level Study and the US Census Bureau’s American Community Survey (ACS), we found that our estimates were reliable and could be used for estimating county-level population health measures (10). Considering the growing needs for local health data at ever-smaller geographic areas, it is necessary to further evaluate our method at subcounty levels. This is important because the method described here was used in an ongoing project, the 500 Cities Project (https://www.cdc.gov/500cities), which provides small-area estimates at the city and census tract levels for a selected set of measures related to public health priorities and impact. In the present study, we selected an independent source of data, the Boston BRFSS, to serve as a benchmark for validating our city-level estimates. Boston BRFSS was designed to collect samples for estimating public health measures that would be representative at the level of the city of Boston, Massachusetts. Additionally, it provided estimates of health measures at neighborhood and zip code levels. Although the survey design did not show how representative the estimates were, the results were adequate for comparison purposes to assess the advantages and disadvantages of our model-based estimates at such levels.

## Methods

### Data sources

The BRFSS is a national, state-based survey of the US adult population aged 18 years or older; it provides valid national and state-level statistics about selected risk behaviors and health conditions. It uses a disproportionate stratified sample design and is administered annually to households with landlines or cellular telephones by state health departments in collaboration with the Centers for Disease Control and Prevention (CDC). In the present study, we selected 10 health indicators from the 2013 BRFSS, which we defined in the same way they were defined in BRFSS (www.cdc.gov/brfss/annual_data/2014/pdf/codebook14_llcp.pdf): binge drinking, current smoking, no leisure-time physical activity, obesity, current asthma, diabetes, high blood pressure (excluding diabetes and high blood pressure that occur only during pregnancy), sleeping less than 7 hours, frequent mental distress, and frequent physical distress. Sleeping less than 7 hours was based on the question, “How many hours of sleep do you get in a 24-hour period?” Frequent mental distress included reporting stress, depression, or problems with emotions for 14 days or more during the past 30 days. Frequent physical distress included reporting having physical illness and injury for 14 days or more during the past 30 days. All outcomes were categorized as binary variables (yes or no). Respondents who had missing values, refused to answer, or answered “did not know” were excluded. The demographic variables were thirteen 5-year age groups (from 18 y to ≥80 y), sex (male and female), race/ethnicity (non-Hispanic white, non-Hispanic black, American Indian or Alaska Native, Asian/Native Hawaiian/other Pacific Islander, other single race, 2 or more races, and Hispanic), and education attainment (<high school diploma, high school diploma, some college, and ≥bachelor’s degree).

The Boston BRFSS, which was administered separately from BRFSS by the Boston Public Health Commission, focused on the health of residents in Boston, Massachusetts. It was conducted in 2010 and 2013 and included many of the same core questions of BRFSS and questions particular to Boston. Boston BRFSS in both years featured a nonoverlapping, dual-frame sample design and was administered to households by random–digit dialing to landline and cellular telephones. In 2010, 3,015 interviews were completed in the landline sample, and 306 interviews in the cellular telephone sample; in 2013, 2,448 interviews were completed in the landline sample and 1,572 interviews in the cellular telephone sample. Boston BRFSS data provided city-level information about the prevalence of selected health risk behaviors as well as knowledge of both health risks and beneficial health behaviors. In our analysis, we combined Boston BRFSS 2010 and 2013 data and defined health indicators in the same way they were described for the BRFSS. The survey included residents of 29 zip code areas (4 zip codes were excluded from calculation of direct survey estimates because they had fewer than 50 respondents, leaving 25 zip code areas included in the present study) and 15 neighborhoods (defined as clusters of adjacent zip codes).

### Statistical analysis

In the 500 Cities Project, we used the MRP modeling framework to estimate the prevalence of the selected health indicators for the 500 largest US cities by US 2010 Census population. Details about the MRP modeling framework can be found in our previous publication (9). Briefly, we constructed multilevel logistic regressions for each indicator:

*P* (*Y_ijk _*_= 1_) = *logit* ^−1 ^(*X_i_β* + *re_j_* + *re_k(j)_*)

where

*Y* is the health indicator that was categorized as yes or no;
*X_i_* is a vector of demographic variables: individual-level age group (reference = 18–24 y), sex (reference = female), race/ethnicity (reference = non-Hispanic white alone), education attainment (reference = less than high school diploma) from the 2013 BRFSS, and county-level percentage of adults below 150% of the poverty line, which was obtained from the 5-year (2009–2013) ACS;
*re_j_* is the state-level random effect; and
*re_k(j)_* is the random effect of county nested in the state.

We used PROC GLIMMIX in SAS version 9.3 (SAS Institute, Inc) to construct the models. The residual pseudo-likelihood estimation method was selected to estimate the model parameters (METHOD = RSPL), and Variance Components was selected as the model’s covariance structure (TYPE = VC). Second, we estimated the prevalence at various geographic levels via post-stratification. We linked estimated parameters from both fixed effects and random effects with the local area population (2010 Census population data) to compute predicted probability of developing a given health indicator (eg, high blood pressure). To obtain prevalence estimates at the city, neighborhood, and zip code tabulation area levels, we aggregated the census-block–level predicted probability to these respective levels.

The US Census Bureau does not publish census-tract–level population data for education attainment by age, sex, and race. To address this issue, we used a bootstrap method to impute individual-level education attainment status during model prediction. This method is detailed elsewhere (11). The census-tract–level percentage of population for education attainment and poverty variables were obtained from the 5-year (2009–2013) ACS. Because we had 1,000 simulation draws, the final estimates were described as the mean small-area estimates (SAEs) and 95% confidence intervals (CIs).

The BRFSS model–based estimates (*m*) for Boston from the 500 Cities Project were assessed by a comparison with direct Boston survey estimates (*s*), which were calculated by using SUDAAN (RTI International) by city, neighborhood (based on zip code), and zip code. For city-level comparison, we assessed the accuracy by observing whether the point estimate fell within the bounds of 95% CIs of the corresponding direct estimates. We calculated relative difference ([*m* – *s*] * 100/s) to indicate whether our estimates underestimated or overestimated the direct estimates. For the neighborhood-level and zip code-level comparisons, we calculated absolute difference (|*m* − *s*|) for each neighborhood and zip code respectively, and tabulated median (interquartile range) for all 15 neighborhoods and 25 zip codes. We measured the accuracy of model-based estimates by using the number and percentage of neighborhoods or zip codes with model-based estimates that were within 95% CIs of corresponding direct survey estimates. The correlation between the 2 sets of estimates was measured by the Pearson correlation coefficient (*r*) and Spearman *ρ* (α = 0.05 was used for statistical significance test). We also made maps of Boston, Massachusetts, by neighborhoods and zip codes to compare geographic patterns in the prevalence of each indicator between the 2 sets of estimates. The model-based estimates and direct estimates were shown on the maps as their quartiles among neighborhoods and zip codes respectively.

## Results

We used data on 483,865 (98.0%) BRFSS 2013 participants from 50 states and the District of Columbia in the multilevel logistic models to obtain model-based estimates for Boston. In Boston BRFSS 2010 and 2013, 7,340 participants (3,320 in 2010 and 4,020 in 2013) were included; item response rates for the selected health indicators were greater than 90%. Data on sleeping and no physical activity were available only in 2013. In the comparison of the means of city-level BRFSS model-based prevalence estimates with direct survey prevalence estimates, the model-based estimates tended to have narrower 95% CIs (Table 1). Among the selected health indicators, the model-based estimates were close to the estimates obtained from the Boston BRFSS survey data for current smoking, no leisure-time physical activity, sleeping less than 7 hours, diabetes, high blood pressure, and current asthma. For example, the model-based prevalence estimate for diabetes (mean, 7.8%) was well within the 95% confidence interval (7.2%–8.7%) of the direct survey estimates (7.9%) from the Boston BRFSS survey. The model-based estimates of 4 health indicators (binge drinking, obesity, frequent mental distress, and frequent physical distress) were not within the bounds of the 95% CIs of the direct survey estimates. Except for binge drinking, the model-based estimates overestimated the prevalence of the 4 health indicators. The biggest discrepancy between the 2 estimates was observed for frequent mental distress (relative difference, 30.6%).

**Table 1:** Direct Boston Behavioral Risk Factor Surveillance System (BRFSS) Estimates and BRFSS Model-Based Estimates for City-Level Prevalence of Selected Health Indicators

Indicator	Prevalence (95% CI)^a^	Relative Difference, %
**Binge drinking**		
Direct survey estimates	25.5 (23.8–27.2)	−7.3
BRFSS model-based estimates	23.6 (23.5–23.7)	
**Current smoking**		
Direct survey estimates	18.7 (17.3–20.3)	0.9
BRFSS model-based estimates	18.9 (18.5–19.2)	
**No leisure-time physical activity**		
Direct survey estimates	22.5 (20.7–24.3)	3.2
BRFSS model-based estimates	23.2 (23.0–23.4)	
**Obesity**		
Direct survey estimates	19.9 (18.6–21.3)	10.6
BRFSS model-based estimates	22.0 (21.9–22.1)	
**Sleep less than 7 hours**		
Direct survey estimates	38.8 (36.6–41.0)	1.8
BRFSS model-based estimates	39.5 (39.3–39.6)	
**Current asthma**		
Direct survey estimates	11.4 (10.3–12.6)	1.6
BRFSS model-based estimates	11.6 (11.5–11.6)	
**Diabetes**		
Direct survey estimates	7.9 (7.2–8.7)	−1.3
BRFSS model-based estimates	7.8 (7.8–7.9)	
**High blood pressure**		
Direct survey estimates	24.3 (23.0–25.7)	1.1
BRFSS model-based estimates	24.6 (24.5–24.7)	
**Frequent mental distress**		
Direct survey estimates	10.4 (9.4–11.4)	30.6
BRFSS model-based estimates	13.5 (13.3–13.7)	
**Frequent physical distress**		
Direct survey estimates	9.1 (8.3–10.0)	21.4
BRFSS model-based estimates	11.0 (10.9–11.2)	

Abbreviations: CI, confidence interval.

a For BRFSS model-based estimates, the prevalence was the mean of estimates.

We also compared model-based estimates with direct survey estimates at the neighborhood level (Table 2). The percentages of neighborhoods with model-based estimates that fell within the bounds of 95% CIs of the corresponding direct estimates ranged from 73.3%-100% (Accuracy in Table 2). The 2 sets of estimates showed strong correlation for binge drinking, no leisure-time physical activity, obesity, sleeping less than 7 hours, diabetes, high blood pressure, and frequent physical distress (no. of neighborhoods = 15; range of Pearson *r* = 0.62–0.89). Correlations were not significant for current smoking (no. of neighborhoods = 15, Pearson *r* = 0.30), current asthma (no. of neighborhoods = 15, Pearson *r* = 0.33) and frequent mental distress (no. of neighborhoods = 15, Pearson *r* = 0.31), which suggests no significant linear relationship between the 2 estimates derived from the 2 approaches. Spearman correlation showed similar results. However, some discrepancies exist between the 2 sets of estimates. First, the model-based estimates suggest a narrower range than that suggested by direct surveys. Second, the interquartile ranges of the absolute differences indicated that the 2 sets of estimates differed across the neighborhoods. Generally, the larger neighborhoods had smaller differences between the 2 sets of estimates than the smaller neighborhoods.

**Table 2:** Distribution of BRFSS Model-Based Estimates and Comparison with Direct Survey Estimates for Prevalence of Selected Health Indicators for 15 Neighborhoods in Boston

Indicator	Estimates						Absolute Difference, Median (IQR)	Accuracy^a^, n (%)	Pearson *r*	Spearman *ρ*
	Min	Q1	Median	Q3	Max	Mean				
**Binge drinking**										
Direct survey estimates	13.8	20.6	23.1	31.2	37.4	25.4	3.4 (4.3)	12 (80.0)	0.84^b^	0.82^b^
BRFSS model-based estimates	17.1	19.7	22.7	26.5	28.2	23.1				
**Current smoking**										
Direct survey estimates	12.6	14.7	16.1	22.5	24.9	18.2	4.3 (3.8)	13 (86.7)	0.30	0.24
BRFSS model-based estimates	16.3	17.3	18.9	19.8	20.6	18.6				
**No leisure-time physical activity**										
Direct survey estimates	9.9	16.8	20.3	26.6	35.1	21.9	4.8 (5.8)	12 (80.0)	0.62^b^	0.57^b^
BRFSS model-based estimates	20.6	21.6	22.7	25.1	26.6	23.3				
**Obesity**										
Direct survey estimates	9.9	14.2	21.2	25.5	33.3	20.3	2.2 (4.5)	11 (73.3)	0.85^b^	0.83^b^
BRFSS model-based estimates	16.0	19.6	21.9	26.0	31.2	22.7				
**Sleeping less than 7 hours**										
Direct survey estimates	27.8	33.0	39.7	41.1	49.1	38.4	2.9 (3.9)	15 (100)	0.66^b^	0.49
BRFSS model-based estimates	35.7	37.4	38.5	42.3	45.0	39.5				
**Current asthma**										
Direct survey estimates	6.3	7.4	11.1	14.7	18.5	11.2	3.3 (2.6)	12 (80.0)	0.33	0.39
BRFSS model-based estimates	10.4	11.0	11.3	12.2	13.3	11.6				
**Diabetes**										
Direct survey estimates	3.2	4.4	7.3	9.3	16.6	8.0	1.2 (1.4)	12 (80.0)	0.89^b^	0.88^b^
BRFSS model-based estimates	3.8	6.1	8.2	10.3	13.0	8.2				
**High blood pressure**										
Direct survey estimates	13.2	21.7	24.0	29.7	39.1	24.8	2.9 (3.5)	13 (86.7)	0.89^b^	0.80^b^
BRFSS model-based estimates	13.8	21.5	25.5	30.5	38.2	25.8				
**Frequent mental distress**										
Direct survey estimates	5.4	9.2	10.4	11.5	14.0	10.2	2.6 (2.2)	12 (80.0)	0.31	0.40
BRFSS model-based estimates	11.3	12.4	13.2	13.6	15.4	13.3				
**Frequent physical distress**										
Direct survey estimates	5.1	6.2	9.2	10.4	14.9	9.1	1.9 (1.3)	12 (80.0)	0.80^b^	0.84^b^
BRFSS model-based estimates	8.8	10.1	11.2	12.3	13.6	11.3				

Abbreviations: BRFSS, Behavioral Risk Factor Surveillance System; IQR, interquartile range; Q1, quartile 1; Q3, quartile 3.

a Number and percentage of neighborhoods with BRFSS model-based estimates that were within 95% confidence intervals of corresponding direct survey estimates.

b *P* < .05.

The prevalence of each indicator varied among neighborhoods (https://image.ibb.co/cD8YJa/17_0281_01a.jpg and https://image.ibb.co/iZPUrv/17_0281_01b.jpg). Yet the 2 sets of estimates differed in identifying the lowest prevalence and the highest prevalence for certain health indicators. For example, the prevalence of diabetes varied consistently by geography between the 2 estimates, whereas the prevalence of current smoking was indicated as lowest in model-based estimates and but modest to highest in the local survey.

A similar pattern between the 2 sets of estimates was observed at the zip code level (Table 3) (https://image.ibb.co/hF13Ja/17_0281_02a.jpg and https://image.ibb.co/gbLDJa/17_0281_02b.jpg). The correlations were moderate to strong for binge drinking, no leisure-time physical activity, obesity, sleeping less than 7 hours, current asthma, diabetes, high blood pressure, and frequent physical distress (no. of zip codes = 25, range of Pearson *r* = 0.53–0.89). Spearman correlation showed similar results. The percentages of zip code areas with model-based estimates that fell within the bounds of 95% CIs of the corresponding direct estimates ranged from 76.0% to 88.0% (Table 3).The 2 sets of estimates had larger differences across the zip codes than neighborhoods. Compared with direct survey estimates, the model-based estimates tended to vary less in the prevalence among 2 or more adjacent zip codes (https://image.ibb.co/hF13Ja/17_0281_02a.jpg and https://image.ibb.co/gbLDJa/17_0281_02b.jpg).

**Table 3:** Distribution of BRFSS Model-Based Estimates and Comparison with Direct Survey Estimates for Prevalence of Selected Health Indicators for 25 Zip Codes in Boston^a^

Indicator	Estimates						Absolute Difference, Median (IQR)	Accuracy^b^, n (%)	Pearson *r*	Spearman *r*
	Min	Q1	Median	Q3	Max	Mean				
**Binge drinking**										
Direct survey estimates	10.7	20.6	25.7	34.4	43.0	27.1	4.6 (7.8)	20 (80.0)	0.62^b^	0.41^b^
BRFSS model-based estimates	16.6	20.5	24.0	27.2	31.0	23.8				
**Current smoking**										
Direct survey estimates	12.6	15.1	17.0	23.4	28.7	18.8	4.3 (2.4)	22 (88.0)	0.27	0.37
BRFSS model-based estimates	16.3	18.1	18.9	19.9	21.1	18.9				
**No leisure-time physical activity**										
Direct survey estimates	2.4	16.8	21.0	23.9	35.1	20.7	4.7 (5.2)	20 (80.0)	0.62^b^	0.66^b^
BRFSS model-based estimates	19.1	21.2	22.6	24.9	28.3	23.1				
**Obesity**										
Direct survey estimates	7.9	10.9	20.0	22.8	33.3	18.2	3.4 (6.1)	20 (80.0)	0.89^b^	0.78^b^
BRFSS model-based estimates	14.5	18.0	20.7	23.6	32.2	21.8				
**Sleeping less than 7 hours**										
Direct survey estimates	16.2	29.6	38.4	41.1	58.3	36.1	4.0 (6.9)	22 (88.0)	0.61^b^	0.51^b^
BRFSS model-based estimates	35.7	37.3	38.5	40.7	45.2	39.3				
**Current asthma**										
Direct survey estimates	1.4	7.6	10.1	13.3	20.1	10.6	3.3 (3.0)	20 (80.0)	0.53^b^	0.60^b^
BRFSS model-based estimates	9.6	11.0	11.3	11.9	13.3	11.5				
**Diabetes**										
Direct survey estimates	0.6	4.1	7.0	9.3	17.0	7.2	1.4 (2.5)	21 (84.0)	0.80^b^	0.59^b^
BRFSS model-based estimates	2.7	6.1	7.8	9.0	13.4	7.8				
**High blood pressure**										
Direct survey estimates	7.2	17.6	22.5	27.6	39.1	22.4	2.0 (3.5)	22 (88.0)	0.83^b^	0.54^b^
BRFSS model-based estimates	11.2	20.4	23.5	28.4	38.2	24.7				
**Frequent mental distress**										
Direct survey estimates	2.7	8.1	10.6	13.2	22.0	10.5	3.1 (4.3)	20 (80.0)	0.16	0.40^b^
BRFSS model-based estimates	11.3	12.4	13.2	14.1	15.8	13.4				
**Frequent physical distress**										
Direct survey estimates	0.8	6.8	8.6	9.6	15.7	8.3	2.0 (3.3)	19 (76.0)	0.70^b^	0.61^b^
BRFSS model-based estimates	7.8	9.7	11.1	11.8	15.1	11.0				

Abbreviations: BRFSS, Behavioral Risk Factor Surveillance System; IQR, interquartile range; Q1, quartile 1; Q3, quartile 3.

a Number and percentage of neighborhoods with BRFSS model-based estimates that were within 95% confidence intervals of corresponding direct survey estimates.

b *P* < .05.

## Discussion

This study compared estimates of 10 selected health indicators generated by the MRP method with direct survey estimates for the city of Boston. For city-level comparisons, the estimates showed strong agreement with the direct estimates for most of the indicators, yet discrepancies were remarkable for frequent mental distress. When comparisons were made at sub-city level, we found that model-based estimates had moderate or strong correlations with direct survey estimates for most indicators; however, depending on the health indicator, there were important differences between the 2 approaches to prevalence estimation.

Many chronic diseases or conditions are affected by individual behavioral factors as well as contextual factors such as geographic location (12). Multilevel models can account for geographic variations by including random effects and have been suggested to be superior to the separate linear regression model for small-area estimation (7,8,13,14). Several applications of multilevel regression models in small-area estimation for chronic diseases or health-related behaviors have been described in recent years (5,6,15,16). Yet such applications require further evaluation because of the lack of external validation. The reason is that few health surveys were designed to generate sub-county estimates for chronic disease and health behavior indicators. Hudson used local administrative hospitalization data to validate estimates of mental disability generated by “regression synthetic estimation fitted using area-level covariates” for zip codes, towns, and cities in Massachusetts and found that Pearson correlation *r* ranged from 0.51 to 0.58 (17). Twigg and Moon compared the neighborhood-level SAEs generated by multilevel models by using a national dataset with local survey health data. Although the results from these local surveys were adequate for comparison, they were not designed for neighborhood-level estimates (18). The Boston BRFSS survey is desirable for the external validation and comparison at the city level because it was originally designed for estimation of city-level chronic health conditions and behaviors; it is contemporaneous with CDC’s BRFSS and used the same survey questions for most of the health indicators. The 2 surveys (2010 and 2013) used the same design methodology and can be combined to obtain a larger sample size; and finally, the Boston BRFSS had high item-response rates.

Although we found good consistencies for most of the selected indicators when comparing our city-level, model-based estimates with direct survey estimates, we found considerable discrepancies for binge drinking, obesity, frequent mental distress, and frequent physical distress, which may be attributable to a few types of bias. For example, a bias toward reporting lower weights in women and higher heights in men is well known in self-reported obesity data and such bias differs by demographic factors (19). Recall bias is more common in reporting health behaviors than in reporting diagnosed chronic diseases. Generally we found a better match between BRFSS model-based estimates and direct survey estimates for diagnosed chronic diseases (diabetes, high blood pressure, and current asthma) than for health behaviors. Frequent mental distress and frequent physical distress are self-evaluated and are not reliable indicators of illness; instead, they are considered to be indicators of self-reported quality of life. Additionally, sample size is a common concern for a local survey, particularly for those indictors with a low prevalence in the population, such as current asthma, frequent mental distress, and frequent physical distress. Finally, the model-based estimation approach may overestimate the prevalence of an indicator if public health interventions that targeted that indicator were implemented at the local level during or just before the survey period. This approach cannot detect or evaluate the effects of local-level interventions.

With regard to the comparisons between the 2 sets of estimates at neighborhood and zip code levels, moderate to strong correlation was observed for most of the indicators, which indicates that higher (or lower) values from one approach match the higher (or lower) values from the other. Yet limitations on each approach should be noted. First, although direct survey estimates are often considered as reliable benchmarks, they are vulnerable to many changes other than the real population changes and tend to overestimate the true ranges of SAEs (19,20). This overestimation became more evident when the area size was smaller. We observed wide ranges at the neighborhood and zip code levels. The extreme rates may reflect the low base denominator number (18). On the other hand, despite the advantages of multilevel modeling, the narrow ranges in model-based estimates may be artificially caused by the modeling process that shrank the highest and lowest rates towards the global mean of the data set (18). Second, the BRFSS model–based estimates had low correlation with direct survey estimates for frequent mental distress and current smoking. Unlike for current smoking, the model-based estimate of frequent mental distress was different from the direct estimate at the city level as well. Thus, besides the reasons we mentioned above, this difference indicates that the model-based estimation may not take into account the complex cross-level variation that is known for frequent mental distress (21).

In summary, our results showed that our methods were able to provide many reliable estimates at the city level. Too often city-level direct estimates, which are preferable, are not available. The modeling approach can be used to meet the growing need for city-level data. Yet the method needs further refinement and assessment for certain health indicators, particularly complex health indicators such as frequent mental distress. At the sub-city levels, given that representativeness was not claimed for the local survey, validation was not easy to achieve. Yet model-based estimation provides useful population health information when high-quality survey data are not available. Our findings suggest further research is needed to identify models that improve reliability of estimates for sub-city geographic areas.
